# Does the Implementation of an Animal Welfare Programme on a Farm Yield a Demonstrable Improvement in Fattening Pig Welfare?

**DOI:** 10.3390/ani12233337

**Published:** 2022-11-29

**Authors:** Patrick Schale, Armin O. Schmitt, Sven Dänicke, Jeannette Kluess, Angelika Grümpel-Schlüter, Engel F. Arkenau

**Affiliations:** 1 Institute of Agricultural Technology, Johann Heinrich von Thünen-Institute, Federal Research Institute for Rural Areas, Forestry and Fisheries, Bundesallee 47, 38116 Braunschweig, Germany; 2 Department of Animal Sciences, Breeding Informatics, Georg-August-Universität Göttingen, Margarethe von Wrangell-Weg 7, 37075 Göttingen, Germany; 3 Institute of Animal Nutrition, Friedrich-Loeffler-Institut, Bundesallee 37, 38116 Braunschweig, Germany; 4 Directorate 82 Digital Innovation, Federal Ministry of Food and Agriculture, Wilhelmstraße 54, 10117 Berlin, Germany

**Keywords:** animal welfare programme, fattening pigs, animal welfare parameters, climate, animal welfare

## Abstract

**Simple Summary:**

Due to social criticism of livestock farming, the “Initiative Animal Welfare” was founded in Germany to improve the conditions in which pigs are kept. At the same time, farmers were obliged to monitor animal welfare parameters which could be recorded directly on the animal, e.g., in the form of tail and ear lesions. Furthermore, there are resource-related animal welfare parameters, e.g., the water flow rate of the drinking troughs, which are to be measured. The aim of this study was to apply animal welfare parameters on farms and compare farms participating in the “Initiative Animal Welfare” with those not participating. The collected data was used to calculate the risk indicated by an animal welfare parameter deviating from the optimum. In addition, an overall score for the farms was calculated from the different animal welfare parameters recorded. It was found that the animal welfare farms did not perform better in terms of both the overall score and the risk of not finding a parameter in the optimum range. One reason for this may be that the farms worked at a very high level in both types of husbandry and thus the improvement in husbandry conditions in the farms with better animal welfare conditions was not measurable.

**Abstract:**

In the course of social criticism of fattening pig farming, an animal welfare programme called “Initiative Animal Welfare” (ITW) was founded to increase animal welfare in pig farming in Germany. Furthermore, there is a legal obligation to record animal welfare parameters as a self-monitoring measure. The “German Association for Technology and Structures in Agriculture” published a guideline on the applicable animal welfare criteria. This guide formed the basis of this study’s data collection. The aim was to apply the animal welfare parameters on farms by comparing the results between farms participating in ITW with those not participating. A cumulative score was calculated by evaluating the collected data. In addition, the relative risk was calculated in order to estimate the risk of finding a negative expression of a parameter. Our data show that ITW farms did not perform significantly better than the farms without ITW in terms of both the cumulative score and the relative risk. Overall, it must be considered that in both farm variants the occurrence of negative evaluations was very rare and the visited farms thus certainly can be considered to be well-managed farms. Climate parameters were recorded in each compartment and showed no significant differences in most cases.

## 1. Introduction

In German society, there is an increasingly critical view of farm animal husbandry, in particular focusing on animal welfare in conventional pig farming. Major points of criticism in pig husbandry are: the available space for each pig, tail docking, the lack of organic enrichment material and bedding as well as outdoor climate stimuli. In addition to current efforts to restructure livestock industry, in 2015 representatives from agriculture, the meat and food retail industry founded the “Initiative Animal Welfare” (“Initiative Tierwohl” (ITW)) with the aim of promoting animal welfare in pig and poultry farming, addressing the raised criticisms [1]. As an incentive to implement ITW-defined animal welfare measures on farms going beyond the legal minimum requirements, pig farmers receive a premium payment per slaughtered pig. These measures consist of basic and optional criteria, differing in the premium paid to the farmers [2].

The first programme phase ran from 2015 to 2017 with a funding of approximately EUR 85 million/year. For each kilogramme of pork or poultry sold, food retailers paid EUR 0.04 to ITW, regardless of whether it originated from ITW farms or not. A total of 14.4 million pigs (suckling piglets, weaned piglets, and fattening pigs), 5.8 million of which were fattening pigs participated in the first programme phase [3].

During the second programme phase from 2018 to 2020, food retailers increased their premium to EUR 0.0625 per kilogramme of pork or poultry sold, thus EUR 132 million became available per year [3]. As of April 2018, 25.3 million pigs, of which 10.7 million were fattening pigs, were participating in the second programme phase of the ITW [1].

In 2021, the third programme phase started, which will run until 2023 [4].

The data for this study was collected during the second programme phase of the ITW. Therefore, this programme phase is described in more detail below.

Farms participating in the ITW are committing themselves to implement basic criteria that are detailed in a German quality assurance system for food, so-called “QS Quality and Security” (“QS Qualität und Sicherheit GmbH”). These QS criteria [5] are supplemented with additional basic factors (Table 1) and farms participating in the ITW programme receive annual compensation as well as a premium per pig. Choosing from the optional criteria of the ITW programme increases the premium accordingly [2].

Since 2014, German farmers were obliged to record animal welfare parameters as part of their own farm inspection [6,7]. However, there are no detailed legal regulations providing the method of recording animal welfare parameters. In order to provide farmers with assistance in recording such welfare parameters, the “German Association for Technology and Structures in Agriculture” (“KTBL: Kuratorium für Technik und Bauwesen in der Landwirtschaft e.V.”) has published a guideline on welfare assessment in pig production [8], hereafter abbreviated as the KTBL guideline.

However, neither ITW criteria nor the KTBL guideline include the compartment’s climate as an important factor contributing to the pig´s well-being. In the cattle sector, the temperature-humidity index (THI) is often calculated, e.g., aiding in the assessment of heat stress. In the pig sector, the THI has hardly been used so far [9,10].

The aim of this study was to assess animal welfare objectively on commercial pig farms either participating or not in the ITW programme (second programme phase), using a combination of the KTBL guideline and environmental parameters. We investigated the question: does the implementation of a voluntary welfare programme such as ITW lead in fact to demonstrably improved welfare in fattening pigs?

## 2. Materials and Methods

Between December 2018 and July 2019, the KTBL guideline was applied to 35 fattening pig farms in Lower Saxony, Germany. The farms were approached from an existing data pool and new farms also agreed to participate through a call or direct approach. Nineteen out of thirty-five farms were participating in the ITW at the time of the farm visit (ITW), and sixteen did not (CONV). Participation in the project was voluntary for the farms and each farm was visited once in the mentioned period. All viewed pigs were kept in forced-ventilated barns.

### 2.1. Data Collection

#### 2.1.1. KTBL Guideline

The KTBL guideline by Schrader et al. [8] includes eight criteria to be assessed per individual pig (Table 2) with either two or three levels. Level 0 indicates that there is no problem. Level 1 in parameters separated in two levels and level 2 for parameters with three levels correspond to the occurrence of a problem. Level 1 in parameters with three levels suggests that the parameter must be further monitored. The guideline specifies the assessment and evaluation frequency for each parameter. This is usually every six months.

In addition to the animal-based parameters, the water flow rate of drinkers was assessed according to the KTBL guideline in order to check whether pigs were sufficiently supplied with water. The required flow rate was differentiated according to the live weight of pigs (Table 3), but did not differentiate between any deviations from the required flow rates. Therefore, this study assigned two levels: level 0 adhering to required flow rates and level 1 for possible deviations (too low/too high). In order to cover all fattening phases in the random selection of pens, animals from all three weight ranges of the drinking flow rates in Table 3 were assessed. For this purpose, the three weight ranges were given live weight classes (lwc) for the later evaluation of the animal-based parameters for the individual fattening phases (Table 3).

#### 2.1.2. Description of Farms

The KTBL guideline specifies a sampling of exactly 150 animals. Since in the present study only the pens to be sampled were randomly selected during the assessment on the farms, all pigs in a pen were always assessed both in the small and big groups. As a result, usually more than 150 pigs were assessed per farm.

In the context of the later evaluation, the data were divided according to the seasons winter, defined as the period from 16 October to 15 April, and summer, defined as the period from 16 April to 15 October. During the winter, twelve ITW farms and twelve CONV farms were visited. In summer, seven ITW farms and four CONV farms were assessed. Each farm was visited only once, resulting in a random distribution between winter and summer.

Table 4 describes the farms separately for ITW and CONV farms. In this study, the barn was defined as a self-contained building, although there could be more than one barn on a farm. There were several compartments in one barn and, in each compartment, there were one or more pens where pigs were kept.

In Germany, the legal standard is 0.75 m^2^/pig with an average group weight of more than 50 kg/pig to 110 kg/pig [12]. On nine out of 16 CONV farms, the stocking density corresponded to the legal standard of 0.75 m^2^/animal, whereas on the other CONV farms, the stocking density was lower with up to 0.91 m^2^/animal. Due to the mandatory criterion of 10% more space on the ITW farms, these farms must provide at least 0.83 m^2^/pig. The average stocking density on the ITW farms was 0.88 m^2^/animal. Three out of nineteen ITW farms matched the legal requirement of 0.83 m^2^/animal stocking density.

With regard to the data on tail length, it must be considered that, due to the randomly selected pens within the context of the assessment, the data refer exclusively to the pens that were assessed. Pens that were not sampled were not included in the count. Consequently, there may be deviations from the real situation on the farms due to the random selection of the sample. It must also be considered that some farms kept pigs with a tail length of at least 2/3 of the original length only in some pens.

On most farms, fattening pigs were group-housed with less than 20 animals per pen.

Pigs were fed via a slop feeding system in the majority of the investigated farms, although there were three farms that had implemented different feeding systems, e.g., the slop and liquid or the dry and slop feeding system.

In Table 4, there may be a larger number of answers in the various categories describing the farms than the number of farms that was specified in the first row. This may be caused by farm expansions or by modification of existing barns. An example of this is the category group size where there were 22 answers in total with only 19 ITW farms, because on some farms there were two configured group sizes.

The enrichment of pens was, on most farms, provided by means of a combination of organic and inorganic materials, e.g., a ball on a chain and a wooden batten. The use of organic or inorganic material as the sole source of enrichment was equally distributed over ITW and CONV farms.

ITW farms had the opportunity to implement additional criteria through the ITW. Fourteen out of nineteen farms took advantage of this. For the criterion of drinking from the open space, the ITW also approves bowl drinkers at the feeder, if they are separated from the feeding area by their design [13]. This possibility was used by three farms. A total of five ITW farms had separated bowl drinkers, but one of them did not use ITW funding. Five farms implemented more than one further criterion.

#### 2.1.3. Conducting the Assessment on the Farms

The pens were chosen at random with approximately the same number of pigs sorted into the three live weight classes in order to cover all fattening phases in the assessment. On farms where all three live weight classes were housed, at least 50 pigs per live weight class were assessed. The animals to be assessed were determined by randomly selecting the pens. In all cases, only pens where the pigs had been housed for at least one week were considered for the assessment, in order to exclude injuries due to ranking fights which could cause a distortion of the results. This was conducted on the basis of the KTBL guideline [8]. The pigs were assessed by the same person on all farms.

When gauging the drinkers, all drinkers in an assessed pen were considered. In the mega groups, the drinkers were randomly selected from the entire pen in proportion to the number of assessed animals in the pen, based on the animal-to-drinker ratio of 12:1 [14], and then checked. The drinkers were left running for 15 s and the water was collected in a litre measure. For data evaluation, the flow rate of the drinkers was then determined for one minute.

In addition to the assessment of the water flow rate and animal-based parameters, indoor climate data were recorded in compartments using a data logger (DK660-0-0-0-5000, Driesen + Kern GmbH, Bad Bramstedt, Germany). During the animal assessment, the logger was placed on feeders or partitioned between pens and recorded the ambient temperature (measuring range: 0–55 °C), relative humidity (measuring range: 0–100%), and carbon dioxide (CO_2_) concentration (measuring range: 0–5000 ppm) of the compartments at one-minute intervals. Climate data were downloaded with the software “InfraLog” (Version 5.7.52 basic; Driesen + Kern GmbH, Bad Bramstedt, Germany). The recording of the climate parameters via the data logger started at the beginning of the assessment. At the beginning of an assessment, the data logger needed 5–10 min to adapt to the ambient temperature in the compartment. During the evaluation of the pigs, the data logger needed 1–2 min to adapt when moved to the next compartment. In order to use only plausible values, solely the last-measured value from each compartment was used for the subsequent data evaluation.

From the measured climate data, temperature (T), and relative humidity (RH), the THI was calculated [15] (Equation (1)):

(1) THI=[(1.8 ∗ T)+32] − [0.55 ∗ (RH/100)] ∗ [((1.8 ∗ T)+32) − 58]

### 2.2. Data Processing and Statistical Analysis

#### 2.2.1. Data Processing

According to the KTBL guideline, animals assigned the worst possible level for a parameter are classified as affected. In the present study, for two-level parameter evaluations, animals with a level of 1 are considered affected, and for three-level parameter evaluations, animals scoring 2 are considered affected. Therefore, for the statistical analysis the levels 0 and 1 of the three-level parameter system were combined to level 0. Adapted to the grading scheme of the two-level parameter system, level 2 became level 1.

Drinkers were considered not to be properly adjusted and thus rated as negative and assigned to level 1 for the statistical analysis, in case the flow rate deviated upwards or downwards from the optimal flow rate as shown in Table 3. If the flow rate did not deviate, the drinkers scored 0, analogously to the animal parameters.

To obtain an overall result, a cumulative score (CS) was calculated. This was calculated for each pig assessed. For this purpose, the individual scores for each animal-related parameter were added up and divided by the number of parameters. Thus, the CS can assume a minimum of 0.000 (all parameters normal) and a maximum of 1.000 (all parameters abnormal). In addition, the percentage frequency of a negative deviation was calculated for each parameter. The mean values and standard deviations were then calculated from the individual values per animal in order to test them for significant differences in a further step.

For the climate data, the mean values and the standard deviations were also calculated from the compartment values collected.

#### 2.2.2. Statistical Analysis

The statistical analyses (parametric and non-parametric tests) were performed using the statistical programme R version 3.6.1 (R CORE TEAM, Wien, Austria) [16] and SAS 9.4 (SAS Institute Inc., Cary, NC, USA, 2016). Differences were considered significant if *p*-value ≤ 0.05. All statistical analyses were conducted once with the tail length parameter and once without.

Fattening pig farmers only exercised a marginal influence on the tail length parameter, as they usually receive piglets with docked tails from the nursery barns (separated production sites). Thus, an evaluation of this parameter scoring 0 meaning no abnormalities was no longer possible in most cases.

##### Climate

The procedure “PROC GLIMMIX” of SAS was used to determine significant differences in the climate parameters temperature, relative humidity, THI, and the CO_2_-content with Equation (2). The farm, the barn, and the compartment were used as covariance parameters. Participation or non-participation in the ITW was defined as a random effect in each case.

In addition to the evaluation of all farms, an evaluation of the climate parameters within the weight classes and for the seasons was also carried out.

(2) $$y_{i}=\beta_{0}+D_{i}+\beta_{1\times 1}+\beta_{2\times 2}+\beta_{3\times 3}+{ɛ}_{i}$$

y_i_ = climate parameter (temperature or relative humidity or THI or CO_2_-content)

β_0_ = regression constant

D_i_ = random effect housing variant (ITW or CONV)

β_1×1_ = linear covariable farm

β_2×2_ = linear covariable barn

β_3×3_ = linear covariable compartment

ɛ_i_ = residual error

##### Parameters

For the parameters, the CS and the percentage distribution were statistically evaluated. Another evaluation of the parameters was the calculation of the relative risk (RR) of the occurrence of problems with the parameters.

The procedure “PROC GLIMMIX” of SAS was also used to evaluate the cumulative score with its percentage deviations for each parameter with Equation (3). The covariance parameters defined here were the farm, the barn, the compartment, the pen, and the pigs. Participation or non-participation in the ITW was likewise applied here as a random effect.

(3) $$y_{i}=\beta_{0}+D_{i}+\beta_{1\times 1}+\beta_{2\times 2}+\beta_{3\times 3}+\beta_{4\times 4}+\beta_{5\times 5}+{ɛ}_{i}$$

y_i_ = CS or percentage distribution of CS for each parameter

β_0_ = regression constant

D_i_ = random effect housing variant (ITW or CONV)

β_1×1_ = linear covariable farm

β_2×2_ = linear covariable barn

β_3×3_ = linear covariable compartment

β_4×4_ = linear covariable pen

β_5×5_ = linear covariable pig

ɛ_i_ = residual error

In order to evaluate each parameter, the RR was calculated with the statistical programme R (Equation (4)). The calculation of the relative risks was conducted for the animal-related parameters at the animal level and for the flow rate of the drinkers at the drinker level. The generated RR indicates how often the analysed parameters in the problematic version occurred in CONV animals compared to ITW animals. If RR = 1, there is no difference between CONV animals and ITW animals. The probability of finding a problem in ITW animals is greater if RR < 1. Accordingly, the probability of finding a problem in CONV animals is greater if RR > 1.

(4) $$\mathrm{RR}=(\frac{a}{\left(a+c\right)})/(\frac{b}{b+d})$$

a = number of animals with problems on a CONV farm,

b = number of animals with problems on an ITW farm,

c = number of animals without problems on a CONV farm,

d = number of animals without problems on an ITW farm.

Fisher’s test was used to determine if the RR value was significantly different from RR = 1. In addition, the confidence interval was calculated to define how much variation lay within the true RR in the base population with a probability of 95%.

The calculation of the RR, the confidence interval, and the determination of significant differences between the ITW and CONV farms was performed in the first step across all farms. In the second step, the collected data were divided and analysed according to the three live weight classes. Furthermore, an analysis was conducted according to the seasons winter and summer (Appendix A, Table A1 and Table A2).

The parameters runts, lameness, and ectoparasites were not analysed by the statistical evaluation of the RR, as they either did not occur at all or only affected a few animals, which would have made the evaluation of the RR meaningless.

## 3. Results

### 3.1. Climate

There were no significant differences to the climatic conditions between ITW and CONV farms, either encompassing all live weight classes or differentiating between the three classes (Table 5). In the evaluation according to the seasons, significant differences could be determined (Table A1).

### 3.2. Animal Parameters

The animal data were analysed using two different statistical methods. Firstly, the CS was evaluated using PROC GLIMMIX and secondly, the RR was calculated. Both variants were evaluated once with the tail length and once without the tail length, whereby the CONV farms kept more pigs with tails that were docked too short.

The CS was significantly lower on the ITW farms compared to CONV farms. This was in particular due to the parameter tail length, on which the fattening pig farmers had only a very small influence. In calculations without the factor tail length, there were no significant differences between the ITW and CONV farms. Both in the calculations with and without the tail length, the CS was at a low level for both variants. Tail length, i.e. a too-short tail, was significantly more often a problem on CONV farms. Another significant difference was observed for lameness (Table 6). However, lameness occurred in only five ITW pigs (one lame pig per farm).

**Figure 1:**
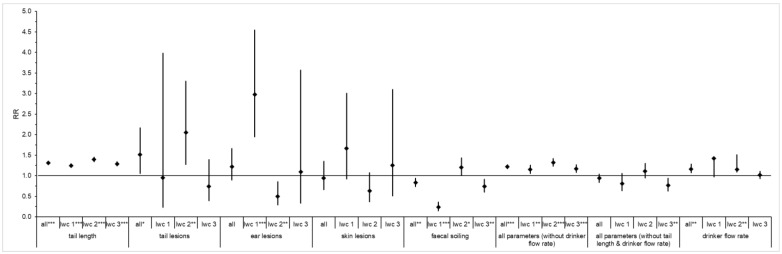
Relative risks (RR, diamond) and the corresponding confidence intervals (vertical lines) for each parameter for all farms and for the respective live weight classes (lwc) (RR = 1 no difference between ITW and CONV farms (horizontal line), if RR > 1 then RR for ITW pigs is lower, if RR < 1 then RR for ITW pigs is higher); number of * denotes significant differences: * = *p*-value ≤ 0.05, ** = *p*-value ≤ 0.01, *** = *p*-value ≤ 0.001.

When looking at the relative risks, it is noticeable that the risk of finding a too-short tail length was significantly lower in the ITW pigs. Concerning tail length, the RR was significantly lower for the ITW pigs when compared to the CONV pigs and when compared in lwc 2. In addition for ear lesions, the risk in lwc 1 was significantly lower on ITW farms, while in lwc 2, the risk was significantly higher. There were no significant differences in skin lesions. In contrast, the risk of being soiled was significantly higher for the ITW pigs, except in lwc 2. When all animal-related parameters are considered together, the ITW farms performed significantly better across the board. If the tail length is left out of the consideration of all parameters, there were no more significant differences, except in lwc 3. In lwc 3, however, the ITW farms performed worse. For the water flow rates, the risk of an incorrect flow rate appeared significantly lower on the ITW farms across all drinkers and in lwc 2 (Figure 1). The relative risks to the seasons are shown in Table A2 in the Appendix A.

## 4. Discussion

When evaluating the CS with all animal-related parameters, a significant difference between the ITW and CONV farms was found, indicating that the ITW farms performed significantly better. Tail length had the most pronounced negative impact on the CS and the CONV farms performed significantly worse than the ITW farms. The results for the relative risk were also comparable. Here, too, an important factor was the tail length parameter, which was highly significant between the ITW and CONV farms. The better performance of the ITW farms was due to the fact that there were more farms within the ITW farms that kept pigs with tails, which have a length of at least 2/3 of the original length. Against the background of Directive 2008/120/EC [17], there must be a significant improvement in this parameter. However, as the fattening pig farmers have no direct influence on the tail length after the delivery of the piglets, if the tail is shorter than 2/3 of the original length, this missing influence must be considered. Therefore, in this study, the CS and the relative risks were also calculated again with all other animal-related parameters except the tail length. Without the latter criterion, there was no significant difference in the CS between the ITW and CONV farms. There were also no differences in the relative risks, except in lwc 3, where even the ITW farms showed a significantly higher risk. One possible reason for the better performance of the ITW farms could be better coordination with the farmer who kept the piglets. For all other parameters, only the lameness parameter showed a significant difference in the percentage distribution between the ITW and CONV farms. This can be explained by the fact that the lameness was only assessed on the ITW farms. However, it must also be considered here that only five pigs were found with lameness, which were then distributed over five farms. The five pigs represented 0.17% of all ITW pigs assessed and thus a very small proportion and therefore is likely to be caused by coincidence.

Looking more closely at the separate relative risks for the tail and ear lesions parameters, the RR for the ITW pigs was lower for tail lesions across all weight classes and in lwc 2, and for ear lesions in lwc 1. In lwc 2, however, the RR for the ITW pigs was significantly higher. The fact that there was no significant difference in tail and ear lesions in the CS can be explained by the fact that in the CS the percentage distribution was used, whereas in the RR the assessment scores were used. One cause of the lesions could be tail and ear biting.

Tail biting is a multifactorial problem on which many different factors have an influence, e.g., space availability, climate, and enrichment material [18]. In this study, the influence of the compartment climate at the time of scoring could be excluded as an influencing factor, as there were no differences between the ITW and CONV farms. On the other hand, a possible influence of the compartment climate before the measuring time on the pigs and thus an influence on the results of the scoring cannot be ruled out, but cannot be reconstructed either. Overall, the temperatures and relative humidity were close to the optimal values of the DIN 18910:2017-08 [19], even if there were isolated cases of exceeding or falling below them.

The consistently larger space availability was an advantage for the ITW pigs. This way, the pigs could better avoid each other in case of stress [20,21,22]. In addition, less stress is to be expected in the feeding and drinking areas due to the lower occupancy and thus the risk of lesions is also lower.

The use of organic materials can also reduce tail and ear biting. According to the TierSchNutztV (2021) [23], enrichment material must be harmless to the pigs´ health and allow the animals to examine, move, and change it so that it serves their exploratory behaviour. Straw, hay, and sawdust in particular, or a mixture of materials, are recognised for this purpose. When this study was conducted, the non-amended TierSchNutztV (2017) [12] still applied and thus the use of materials for animal housing had not yet been specified in the TierSchNutztV (2017) with regard to changeability. However, the “Lower Saxony State Office for Consumer Protection and Food Safety” (Niedersächsisches Landesamt für Verbraucherschutz und Lebensmittelsicherheit” (LAVES)), as the competent authority in Lower Saxony, had already conducted an assessment of enrichment material in 2015 and pointed out that only organic enrichment material fulfilled all the required properties. Thus, at the time of the assessment, all farms, irrespective of whether they were ITW or CONV farms, would have had to use organic enrichment material, such as straw, hay or ropes from natural fibres [24].

For the parameter of faecal soiling, the risk for an occurrence of soiled ITW pigs was significantly higher across all animals and in lwc 1 and lwc 3. In lwc 2, the situation was reversed. Since pigs can only sweat minimally, they seek cooling opportunities for thermoregulation at high temperatures [25]. To prevent hyperthermia, pigs wallow in mud [26]. Since wallowing areas are usually not provided on conventionally managed farms, leading pigs to lie down in the manure area to cool down [8]. This then leads to clearly soiled animals in a poor housing climate [27]. However, all four climate parameters (temperature, relative humidity, THI, and CO_2_-concentration) did not show any significant differences across all farms or in the three live weight classes. The lower stocking density, especially on the ITW farms, may also have meant that the faeces did not pass through the slatted floor that well. This may have led to more faeces in the pens than in pens where the pigs only had the minimum legal space available. Another explanation can be found in possible temperature variations during the day. Since the farm visits usually took place in the morning, it can be assumed that temperatures rose more during the day, especially in summer. This could have led to the pigs lying down more often to cool down. It is unclear why the RR in lwc 2 for faecal contamination was significantly higher for the CONV pigs. This cannot be conclusively clarified from the available data.

In terms of water supply, the ITW farms performed significantly better on the RR in weight class 2 and when considered across all weight classes. This may also have made a positive contribution to the fewer lesions in the ITW pigs.

In addition to the water quality, the flow rate of the drinking troughs must be sufficient for the animals [23]. The gradation used in the KTBL guideline is difficult to implement in practice. Here, the drinkers are usually adjusted before the fattening period and only checked for function during that time. Since the guideline is continuously reviewed and further developed, the second edition of the KTBL guideline now also only specifies a flow rate of 0.8–1.8 L/min for the entire fattening period [28]. This is also much more practicable in practice than the previous gradation into three weight ranges.

In particular, flow rates that are too low can lead to inadequate water intake and thus leads to thirst and discomfort. Water is essential for many bodily functions [29]. According to Botreau et al. [30], the absence of permanent hunger and thirst is a fundamental prerequisite for the well-being of pigs and is also required by animal welfare legislation [6]. Unwellness leads to stress, which in turn can lead to tail and ear biting. Too high a flow rate can also make water intake more difficult [31]. It does, however, not lead to the pigs having problems with water intake. Pool drinkers show a clear advantage since pigs can take up water from an open water surface. Furthermore, water intake from an open water surface corresponds to the natural behaviour of pigs, as they are suckling drinkers [32].

Overall, however, flow rates that are too low are more serious from the point of view of animal welfare than flow rates that are too high, as the animal has hardly any opportunity to take up sufficient water with a flow rate that is too low. The KTBL guideline should pay more attention to this, as a flow rate that is too low can be relevant to animal welfare. The animal farmer must ensure a sufficient supply of feed and water [23].

The cumulative scores for the ITW and CONV farms were at a very low level. It is difficult to make a clear statement on the individual parameters, as there were inconsistent results between the weight classes within the parameters. There were also sometimes considerable external influences and the diversity of variants on the farms also played a major role. Due to the small number of farms, this led to a sometimes-strong positive evaluation, such as in the case of tail length. In addition to the different conditions on the farms and thus a large number of influencing factors that were included in this study, the farmers and their management are also essential factors for successful fattening pig husbandry, both in economic terms and with regard to the successful implementation of animal welfare measures. The relatively small sample size also contributes to the fact that environmental effects have a greater impact and thus actual improvements in animal welfare may have been masked. Furthermore, all farms participated in the study voluntarily. In particular, those voluntarily attending farms are often very interested in improvements and in most cases have their animal husbandry at a good to very good level overall, so that strong negative abnormalities are not to be expected. The KTBL guideline does not currently provide instructions for the aggregation of the results of the pigs´ inspection. This complicates a comparison between different self-monitoring farms or a comparison within a farm. In the study by Pfeifer et al. [33] which used the same KTBL guideline, the surveyed farmers also stated that an overall result would help them with valuations and comparability with other farms or comparability with previous animal welfare assessments. Therefore, in this study, the individually scored criteria were combined into a so-called cumulative score as an overall measure. A CS is very helpful for both farmers and veterinary authorities during inspections: it allows for horizontal (between different farms) as well as vertical (within a farm) comparisons and thus gathers information on performance and previous assessments. At the same time, the veterinary authorities obtain the possibility to control farms in a risk-oriented way, if certain farms repeatedly show high negative values. The KTBL guidelines leave out one very important point. Since the majority of fattening pigs in the Federal Republic of Germany are kept in closed, forcibly ventilated barns, the aspect of ventilation is a very important one. A poor indoor climate can have a considerable influence on the animals’ well-being.

In the study by Pfeifer et al. [33], 70% of the farmers (n = 40 farmers surveyed) suggested further parameters that should be added to the KTBL guideline. Among these, barn climate factors, such as air quality, pollutant gas concentration, and temperature, were mentioned most frequently (12 mentions) as key criteria for pig welfare.

So far, there are hardly any simple, practicable ways for farmers to check their housing climates with regard to air temperature and relative humidity. In addition to the air temperature, the relative humidity also plays an important role in the well-being of the pigs. First approaches exist with the use of the THI in which both parameters are calculated with each other via a formula and thus facilitate an overall statement on the barn climate. There is a need for further research here, as the THI has so far mainly been used in the cattle sector and rather rarely in the pig sector [9,10].

In conclusion, participation in the ITW programme was not clearly reflected as having an improvement on pig welfare criteria. Due to the great variability in practice and the small sample size, it is not possible to transfer the results of this study globally to fattening pig farming in Germany. For a more targeted statement, a larger data basis would be absolutely necessary, so there is still a need for research here. Nevertheless, the ITW makes an important contribution to the further development of animal husbandry in Germany. Without secure funding, the implementation of animal welfare measures would be difficult for farmers to realise, as they usually cannot currently generate any additional revenue for their animal welfare measures.

## 5. Conclusions

Applying animal welfare parameters as recommended by the KTBL guideline, this study could not discern an objective difference in animal welfare on farms participating in the ITW programme as compared to conventional farms. The voluntary participation of farmers has certainly played a role in this result as those participants were most likely farms having their animal husbandry at a high level and were actively concerned with animal welfare improvement. This might be proved differently in a randomized selection of farms by the authorities.

Regarding the animal welfare guideline, a cumulative score (CS) of those KTBL parameters would aid farmers with evaluations and comparability with other farms or with previous animal welfare assessments. Furthermore, such a CS could be a useful tool for veterinary authorities during inspections and enable them to control farms in a risk-oriented way. Expanding this CS also on indoor climate characteristics could further improve its usefulness and validity for pig farmers.

## Figures and Tables

**Table 1:** Criteria of the animal welfare programme ITW during the second programme phase [2].

Basic Criteria	
basic criteria of QS Quality and Security	EUR 500.00/year basic contribution
QS-antibiotic monitoring	
QS-indexed slaughter inspection programme	
barn climate check	
drinking water check	
daylight	
additional organic enrichment material	
10% more space	
**fulfilment of all basic criteria**	**EUR 3.30/slaughtered pig**
**optional criteria**	
20% more space	EUR 1.20/slaughtered pig
permanent access to roughage	EUR 1.80/slaughtered pig
body scratching device	EUR 0.60/slaughtered pig
air-cooling system	EUR 0.20/slaughtered pig
drinking from a bowl drinker or open drinker	EUR 0.70/slaughtered pig
**fulfilment of all optional criteria**	**EUR 4.50/slaughtered pig**
**maximum amount paid per pig (basic + optional)**	**EUR 5.10/slaughtered pig**
The above-mentioned sums were additionally paid as a premium on the sales revenue normally generated. The premium was limited to a maximum of EUR 5.10/slaughtered pig.	

**Table 2:** Animal-based parameters according to the KTBL guideline [8,11].

Animal Welfare Indicator	Score or Category	Description
tail length	0	original length
	1	remaining tail length ≥ 2/3 of the original length (i.e., maximum one third of the original length is missing)
	2	remaining tail length < 2/3 of the original length (i.e., more than one third of the original length is missing)
tail lesions	0	tail without clearly visible bleeding wounds, scabs or swellings
	1	tail with clearly visible bleeding wound, scab or swelling
ear lesions	0	ear without clearly visible bleeding wounds and scabs or ear with only linear scratches on the outer side
	1	clearly visible, mostly bleeding wounds and scabs on the ear (especially on the tip, rim or base of the ear)
skin lesions (expect tail and ears)	0	<4 linear lesions with ≥5 cm and no circular lesion with a diameter of ≥2.5 cm (2-euro coin)
	1	4–15 linear lesions with ≥5 cm in and no circular lesions with a diameter of ≥2.5 cm (2-euro coin)
	2	>15 linear lesions with ≥5 cm in or one circular lesion with a diameter ≥2.5 cm (2-euro coin)
manure on the body	0	“unsoiled”: <10% of the surface with faecal deposits
	1	“slightly soiled”: 10 to 30% of the surface with faecal deposits
	2	“severely soiled”: >30% of the surface with faecal deposits
lameness	0	“no or slight lameness”: normal gait (fluent gait, equal stride length, and even weight bearing on all four limbs) or slight lameness (stiff gait, shortened stride length, and increased spinal segment movement)
	1	“severe lameness”: minimum weight bearing on the affected limb, quick alternation between weight bearing and no weight bearing on the affected limb (“tipping”) up to no weight bearing on the affected limb or inability to stand up or to walk
runts	0	normal conditions
	1	animals showing at least two of the four described signs: (1) significantly smaller body size compared to pen mates (2) prominent spine (3) sunken flanks (4) long bristles
signs of ectoparasites	0	no signs of ectoparasites
	1	(1) lice or their eggs: lice and/or their eggs visible to the naked eye, sticking to the bristles, in addition often intense rubbing against objects or scratching with legs or (2) incipient mange: skin irritations, such as many red dots distributed over the body, in addition intense rubbing against objects or scratching with legs or (3) mange: grey-brown crusts on ears, neck, base of the tail or mid-foot area, in addition intense rubbing against objects or scratching with legs

**Table 3:** Evaluation scheme for flow rates of drinkers according to the KTBL guidelines (according to Schrader et al. [8]).

Weight Range of Pigs	Required Flow Rate [L/min]	Live Weight Class (LWC)
up to 50 kg	0.6–1.0	1
51–80 kg	0.8–1.2	2
81–120 kg	1.5–1.8	3

level 0 = measured flow rate in accordance with required flow rate; level 1 = measured flow rate deviating from the required flow rate (higher or lower).

**Table 4:** Description of housing conditions on ITW and CONV farms.

		ITW Farms	CONV Farms
	number of farms	19	16
fattening pig capacity [no. pigs/farm]	median (min–max)	1200 (400–2000)	1004 (420–1824)
stocking density * [m^2^/pig]	median (min–max)	0.84 (0.83–1.09)	0.75 *^1^ (0.75–0.91)
assessed pigs [no. of randomly selected pigs to be assessed]	total	2968	2651
	lwc 1 (up to 50 kg)	939	590
	lwc 2 (51–80 kg)	1166	1114
	lwc 3 (81–120 kg)	863	947
management of tail length *^2^ [no. farms]	original length – tail length ≥ 2/3 of original length	11 *^3^	5 *^3^
	Tail length < 2/3 of original length	19	16
group size [no. farms]	small group (<20 pigs/pen)	17	13
	large group (20–60 pigs/pen)	4	8
	mega group (>60 pigs/pen) *^4^	1	1
floor [no. farms]	fully slatted floor	19	16
	partially slatted floor	0	2
measured drinkers [no. of randomly selected drinkers to be tested]	total	454	322
	lwc 1 (up to 50 kg)	129	75
	lwc 2 (51–80 kg)	173	124
	lwc 3 (81–120 kg)	152	123
type of drinker [no. farms]	nipple drinker	19	16
	bowl drinker, open drinker	5	0
feeding system [no. farms]	dry feeding system	6	2
	slop feeding system	11	9
	liquid feeding system	5	5
enrichment material [no. farms]	organic and inorganic	14	11
	only inorganic	4	5
	only organic	1	0
selected optional criteria of ITW-farms [no. farms]	20% more space	2	/
	permanent access to roughage	4	/
	body scratching device	2	/
	air cooling systems	5	/
	drinking from bowl drinker, open drinker	7	/
	no additional optional criteria	5	/

lwc = live weight class; * legal minimum standard: 0.75 m^2^/pig (mean live weight 51–110 kg/pig) [12]; *^1^ one missing value (One farmer did not give any information on stocking density.), n = 15 farms; *^2^ The data refer exclusively to the assessed pens. Pens that were not assessed were not included in the count. *^3^ ITW and CONV farms, three each, which each only had between one and three assessed pigs in this category. *^4^ pen = compartment; pigs were assessed analogously to the three live weight classes also, whereby the selection of pigs in the mega group was randomized. This was performed by walking through the entire pen and randomly selecting the pigs to be assessed from the entire pen. Each mega group corresponded to a live weight class. On the two farms with the mega groups, one farm had two mega groups, so that pigs from both groups were assessed proportionally. On the other farm, there were a total of six mega groups, of which pigs from three groups were assessed proportionally.

**Table 5:** Indoor climatic conditions in pig compartments on ITW and CONV farms. Data are presented as means ± standard deviation and statistically analysed using PROC GLIMMIX (SAS).

	ITW Farms	CONV Farms	*p*-Value
ambient temperature [°C]	22.5 ± 2.6	19.9 ± 3.5	0.064
relative humidity [%]	63.2 ± 8.7	67.0 ± 10.0	0.138
THI	67.5 ± 3.2	64.3 ± 4.3	0.065
CO_2_-concentration [ppm]	2098 ± 748	1895 ± 476	0.991
n [compartments]	85	71	
lwc 1 (up to 50 kg)			
ambient temperature [°C]	23.0 ± 2.2	19.7 ± 3.0	0.255
relative humidity [%]	69.1 ± 6.7	71.1 ± 11.6	0.627
THI	67.6 ± 2.8	63.9 ± 3.8	0.289
CO_2_-concentration [ppm]	2587 ± 768	2071 ± 511	0.991
n [compartments]	23	18	
lwc 2 (51–80 kg)			
ambient temperature [°C]	22.3 ± 2.8	20.0 ± 3.8	0.235
relative humidity [%]	59.4 ± 8.9	67.2 ± 7.3	0.100
THI	67.5 ± 3.5	64.4 ± 4.4	0.173
CO_2_-concentration [ppm]	1804 ± 593	1902 ± 557	0.995
n [compartments]	36	27	
lwc 3 (81–120 kg)			
ambient temperature [°C]	22.4 ± 2.6	19.9 ± 3.6	0.158
relative humidity [%]	63.1 ± 7.2	63.9 ± 10.4	0.495
THI	67.4 ± 3.3	64.5 ± 4.6	0.160
CO_2_-concentration [ppm]	2073 ± 725	1765 ± 303	0.994
n [compartments]	26	26	

**Table 6:** Cumulative score of animal-based welfare parameters and parameter distribution in ITW- and CONV-housed pigs according to the KTBL guideline. Data are presented as means ± standard deviation and statistically analysed using PROC GLIMMIX (SAS).

	Prevalence [%]			CS with Tail Length		CS without Tail Length	
Parameter	ITW Farms	CONV Farms	Unit	ITW Farms	CONV Farms	ITW Farms	CONV Farms
no. pigs	2968	2651	CS	0.123 ^a^ ± 0.078	0.150 ^b^ ± 0.058	0.034 ± 0.065	0.031 ± 0.064
tail length	74.83	98.34	% of CS	9.354 ^a^ ± 5.426	12.293 ^b^ ± 1.597	/	/
tail lesions	1.72	2.60		0.215 ± 1.625	0.325 ± 1.991	0.245 ± 1.857	0.372 ± 2.275
ear lesions	2.56	3.13		0.320 ± 1.975	0.391 ± 2.177	0.366 ± 2.257	0.447 ± 2.488
skin lesions	2.16	2.04		0.270 ± 1.816	0.255 ± 1.766	0.308 ± 2.075	0.291 ± 2.018
faecal soiling	16.95	14.11		2.118 ± 4.690	1.763 ± 4.352	2.421 ± 5.360	2.015 ± 4.974
runts	0.07	0.04		0.008 ± 0.324	0.005 ± 0.243	0.010 ± 0.371	0.005 ± 0.277
lameness	0.17	0.00		0.021 ^a^ ± 0.513	0.000 ^b^ ± 0.000	0.024 ^a^ ± 0.586	0.000 ^b^ ± 0.000
ectoparasites	0.00	0.00		0.000 ± 0.000	0.000 ± 0.000	0.000 ± 0.000	0.000 ± 0.000

CS minimum: 0.000, CS maximum: 1.000; lines with different letters ^a,b^ in the column CS with tail length differ significantly, *p* ≤ 0.05; lines with different letters ^a,b^ in the column CS without tail length differ significantly, *p* ≤ 0.05.

## Data Availability

The data are not publicly accessible, as this is not possible for data protection reasons. There are no declarations of consent from the farmers, or the farmers refused to allow the data to be publicly accessible, even in anonymised form.

## Appendix A

**Table A1:** Compartment climate in the winter (16 October–15 April) and in the summer season (16 April–15 October). Data are presented as means ± standard deviation and statistically analysed using PROC GLIMMIX (SAS).

Winter	ITW Farms	CONV Farms
ambient temperature [°C]	21.4 ^a^ ± 1.6	18.5 ^b^ ± 2.1
relative humidity [%]	65.0 ± 8.2	68.3 ± 10.1
THI	66.0 ^a^ ± 1.9	62.5 ^b^ ± 2.4
CO_2_-concentration [ppm]	2408 ± 675	1997 ± 435
n [compartments]	57	57
**summer**		
ambient temperature [°C]	24.8 ± 2.7	25.7 ± 1.6
relative humidity [%]	59.5 ± 8.8	61.7 ± 7.6
THI	70.5 ± 3.3	71.4 ± 2.3
CO_2_-concentration [ppm]	1468 ± 428	1479 ± 410
n [compartments]	28	14

Lines with different letters ^a,b^ differ significantly, *p* ≤ 0.05.

**Table A2:** Relative risk, p-value, and confidence intervals for the parameters in the winter (16 October–15 April) and summer season (16 April–15 October).

Parameter	Relative Risk		*p*-Value		Confidence Interval	
	Winter	Summer	Winter	Summer	Winter	Summer
tail length	1.42	1.18	<0.001	<0.001	1.37–1.46	1.15–1.21
tail lesions	1.58	0.99	0.029	1.000	1.05–2.39	0.44–2.22
ear lesions	1.03	1.75	0.929	0.127	0.73–1.45	0.86–3.56
skin lesions	0.47	6.26	<0.001	<0.001	0.30–0.73	2.72–14.39
faecal soiling	0.85	1.03	0.064	0.728	0.71–1.01	0.87–1.22
all parameters (without drinker flow rate)	1.29	1.18	0.095	<0.001	1.22–1.37	1.10–1.28
drinkers flow rate	1.08	1.42	<0.001	0.126	1.06–1.30	0.98–1.34

Winter: n = 1867 ITW pigs and 2023 CONV pigs (calculation at animal level); summer: n = 1101 ITW pigs and 628 CONV pigs (calculation at animal level); winter: n = 310 ITW drinkers and 238 CONV drinkers (calculation at drinker level); summer: n = 144 ITW drinkers and 84 CONV drinkers (calculation at drinker level); *p* ≤ 0.05; winter: n = 1867 ITW pigs, 2023 CONV pigs and 310 drinkers by ITW farms, 238 drinkers by CONV farms; summer: n = 1101 ITW pigs, 628 CONV pigs and 144 drinkers by ITW farms, 84 drinkers by CONV farms.
